# What editors, reviewers, researchers and librarians need to know about the PRESS, MECIR, PRISMA and AMSTAR instruments with regard to improving the methodological quality of searches for information for articles

**DOI:** 10.1590/1516-3180.2020.138625112020

**Published:** 2020-12-21

**Authors:** Maria Eduarda dos Santos Puga

**Affiliations:** I MSc, PhD. Librarian, Evidence-Based Health Program, Universidade Federal de São Paulo (UNIFESP), São Paulo (SP), Brazil; Director, Coordenadoria da Rede de Bibliotecas UNIFESP (CRBU), São Paulo (SP), Brazil; and Information Specialist, Cochrane Brazil, São Paulo (SP), Brazil.; II MD, PhD. Full Professor and Head of the Discipline of Emergency Medicine and Evidence-Based Medicine, Universidade Federal de São Paulo (UNIFESP), São Paulo (SP), Brazil; and Director of Cochrane Brazil, São Paulo (SP), Brazil.

The question that people involved in scientific information and publishing keep asking is “What can we do to further improve the quality of scientific publications?”

Scientific publications contain text that reports on the steps taken within scientific research. The published text is the end product from this work, which deserves to be reported properly and in detail.

Evaluative instruments through which syntheses and synopses of evidence are made add rigor and methodological quality to published studies at all stages, so that the final product will have reliable and reproducible results.

Therefore, in answer to the initial question, we can survey the instruments available to aid in searching for information. A search for information forms an important methodological stage in any scientific investigation, and not just in studies that have the aim of producing a synthesis of the evidence.

The structured tools that are used in assessments and in producing certain types of study such as systematic reviews, technological healthcare evaluations, scoping reviews, rapid systematic reviews, overviews, integrative reviews, and so on, may form instruments that guide editors, reviewers, researchers and librarians. One such instrument was specifically created to guide librarians in evaluating and conducting high-sensitivity search strategies.

Four instruments fall into this category, as follows:

- **MECIR** - Methodological Expectations for Cochrane Intervention Reviews;
- **PRISMA** - Preferred Reporting Items for Systematic Reviews and Meta-Analyses;
- **AMSTAR** - Assessing the Methodological Quality of Systematic Reviews;
- **PRESS** - Peer Review of Electronic Search Strategies.^1^^-^^9^

In Table 1, we present these four instruments for conducting sectional assessments and analyses, specifically for searching for information and developing a search strategy. Through this, it can be seen that the PRESS and MECIR instruments provide more detail for conducting searches than do PRISMA and AMSTAR, including provision of detailed guidance for this stage and greater rigor.^1^^-^^9^

**Table 1:** Instruments used for conducting sectional syntheses of evidence and assessing their quality, in order to evaluate search strategies and select databases^1^^-^^9^

**MECIR** – **METHODOLOGICAL EXPECTATIONS FOR COCHRANE INTERVENTION REVIEWS -** https://methods.cochrane.org/methodological-expectations-cochrane-intervention-reviews. This instrument is used by the Cochrane Collaboration to ensure the rigor and quality of its publications.	
What is MECIR? It consists of methodological standards to which all Cochrane protocols, reviews and updates must adhere, and rules for conducting them and making reports, etc.	
**Searching for studies (C24-C38)** C24. Searching in general bibliographic databases (MEDLINE, Embase) and CENTRAL – **Mandatory** C25. Searching in specialized bibliographic databases (CINAHL, LILACS, PsycINFO) – **Highly desirable** C26. Searching for different types of evidence: specific eligibility criteria regarding the design of the study, to address adverse effects, economic issues or qualitative research issues – **Mandatory** C27. Searching for trial registrations: Investigation of registration of studies and repositories of results, when relevant to the topic, through ClinicalTrials.gov, the WHO International Clinical Trial Registration Portal (ICTRP) and other sources as appropriate – **Mandatory** C28. Searching the grey literature: Investigation of relevant sources of grey literature, such as reports, dissertations, theses, databases and conference abstract databases *–* **Highly desirable** C29. Searching for other comments: Investigation of previous analyses on the same topic – **Highly desirable** C30. Searching reference lists: Verification of reference lists in the studies included and any relevant systematic reviews that were identified – **Mandatory** C31. Investigation of contacts with relevant individuals and organizations: Contacts with relevant individuals and organizations to obtain information on studies that are unpublished or in progress – **Highly desirable** C32. Structuring of search strategies for bibliographic databases: The structure of the search strategies in bibliographic databases around the main concepts of the review should be informed, using appropriate elements from PICO (problem-intervention-comparison-outcome) and the study design. In structuring the investigation, sensitivity should be maximized while seeking reasonable precision. Correct use of the operators “AND” and “OR” should be ensured – **Mandatory** C33. Development of research strategies for bibliographic databases: Appropriate controlled vocabulary needs to be identified (for example, MeSH or Emtree, including “exploded” terms), along with free-text terms (for example, considering spelling variations, synonyms, acronyms, stem operators and proximity) *–* **Mandatory** C34. Use of search filters: Specially designed and tested search filters should be used when appropriate, including highly sensitive Cochrane search strategies for identifying randomized clinical trials in MEDLINE. However, filters should not be used in prefiltered databases. For example, randomized trial filters should not be used in CENTRAL and systematic review filters should not be used in DARE *–* **Highly desirable** C35. Restrictions on database searches: The use of any restrictions in search strategies regarding publication date and publication format needs to be justified – **Mandatory** C36. Documenting the search process: The search process should be documented with sufficient detail to ensure that it can be reported correctly in the review – **Mandatory** C37. Doing searches again: The searches in all the relevant databases should be done again within the last 12 months before the review is published or updated, to check for any results from potentially eligible studies – **Mandatory** C38. Incorporation of discoveries from repeated searches: Any studies identified through repeating or updating the search within the last 12 months before the review is published or updated should be incorporated in full – **Highly desirable**	
**PRISMA – Preferred Reporting Items for Systematic Reviews and Meta-Analyses** - http://www.prisma-statement.org/. This is a checklist for the main recommendations and items to be included in reporting on a systematic review. It relates only to information searches.	
**Information sources:**	
**ITEM 7**: Describe all the information sources in the search (for example: database with dates of coverage or contact with authors to identify additional studies) and the date of the last search.	
**ITEM 8**. Present a complete electronic search strategy for at least one database, including any limits used, so that it can be repeated. Detailed description of the information flow in the different phases of the systematic review (PRISMA flow diagram).	
**AMSTAR 2 – ASSESSING THE METHODOLOGICAL QUALITY OS SYSTEMATIC REVIEW** - https://amstar.ca/Publications.php. This is a critical assessment tool that is used to evaluate the quality of systematic reviews on randomized studies and also, in this version 2, non-randomized healthcare intervention studies.	
**Question 4. Did the review authors use a comprehensive strategy for searching the literature?** They searched at least two databases (that were relevant to the research question) They supplied keywords and/or search strategies They justified any publication restrictions (for example, language) They investigated reference lists or bibliographies in the studies included They investigated registers of trials and studies They included or consulted specialists within the field They investigated the grey literature when this was relevant They did a search within 24 months after concluding the review	
**PRESS** 2015 – Guidelines and recommendations for librarians’ practices^8^	
Here, we highlight the recommendations for librarians, in addition to those in Table 3, which shows the simplified list of PRESS.	
1. Translation of the research question: Assess whether the research question was translated correctly, within the research concepts.	Ideally, the primary search strategy should be submitted to peer review to ensure conceptual precision. The research question, which is normally formatted in accordance with some variation of PICO and fine points about how the research was informed by the reference interview, should be sent with the research strategy.
2. Boolean and proximity operators: Assess whether the elements relating to the research question were combined correctly using Boolean and/or proximity operators.	Look again at the search regarding any instances of errors in Boolean operators. For example, OR may have been accidently replaced by AND (or vice versa), or AND may have been used to link phrases or words (for example, as a conjunction) instead of as a Boolean operator. Note that where NOT was used, there is the possibility of unintentional exclusions, and another device (for example, use of a subject title, verification label or limit) may produce an equivalent result. Check that any use of nesting between square brackets is logical and has been applied as necessary. Also, note whether use of a proximity operator (adjacent, near, within) instead of AND might increase the precision. If proximity operators have been used, consider whether the width chosen is narrow enough to capture all the foreseen instances of the search terms, which may vary depending on whether the database investigated does or does not recognize stop words. Consider whether the width is too broad. If there are restrictions (for example, human populations or elderly populations), check whether an appropriate construction was used.
3. Subject headers (specific for the database): Assess whether there is enough scope in selecting subject headers for the recall to be optimized.	Examine the following elements used in subject titles: absent or incorrect titles, relevance or irrelevance of terms and correct use of explosion for including more restrictive relevant terms. Consider using floating subtitles: in most cases, this is preferable to using subtitles attached to specific subject titles (for example, in MEDLINE, “Neck Pain/and su.fs.” instead of “Neck Pain/su”). Note that subject titles and subtitles are specific to databases.
4. Search for text words (free text): Assess whether the search terms without adequate coverage of the subject title are well represented by free-text terms and whether additional synonyms and antonyms (opposites) and related terms are needed.	Free-text terms are normally used to cover subject headers of absent databases. Consider whether elements using free text might be too narrow or too broad, what the relevance of these terms is and whether synonyms and antonyms have been included.
5. Spelling, syntax and line number: Assess the correctness of the spelling and syntax and the implementation of correct searches.	Review the search strategy for words with spelling mistakes and system syntax errors that are not easily found through spellcheckers. Check each line number and combinations of line numbers to ensure that the logic of the search has been correctly implemented.
6. Limits and filters: Assess whether the limits used (including filters) are appropriate and have been correctly applied.	Review the search strategy to see whether limits that are not relevant for the eligible study designs or for the clinical question were applied, since this could introduce epidemiological bias. Check whether the methodological filters for the search were applied correctly: for example, to ensure that systematic reviews of economic evaluations are not restricted to clinical trials.

## MECIR

The librarian of the Cochrane Collaboration, who has the title of Cochrane Information Specialist (CIS), has the task of **designing and implementing search strategies**. This involves the entire process of defining the question, identifying the vocabulary that covers this question, transcribing the question into a search strategy, selecting the databases, transcribing the strategy for all the databases that were selected (mandatory, specialized and recommended databases), testing the performance of the strategy, adjusting it and running it in all the databases selected for the question. The librarian assists in saving and guiding the management of results obtained through automated systems for selecting and identifying duplicated studies.^1^^,^^2^

The CIS has to ensure that the research methods are documented in accordance with the MECIR standards. These also serve as a compass for the CIS in conducting the whole process.^1^^,^^2^

Involvement of this specialist adds significantly to improvement of the reporting of the research methods and also to evaluation of the general quality of the development process and presentation of the review.

Information specialists’ involvement in traditional research tasks is always recommendable as a central methodological tenet for producing high-quality systematic reviews. However, these professionals’ experience is increasingly being implemented in new ways.

In 2014, The Lancet, one of the world's most important medical journals, published a series of articles on how to improve research and reduce waste within it. These articles are available with open access and are listed in the following Table 2.^10^^-^^17^

**Table 2:** Lancet Reward (REduce research Waste And Reward Diligence) Publications

**Comments**
How should medical science change?^10^ Biomedical research: increasing value, reducing waste.^11^
**Series (2014)**
How to increase value and reduce waste when research priorities are set.^12^ Increasing value and reducing waste in research design, conduct, and analysis.^13^ Increasing value and reducing waste in biomedical research regulation and management.^14^ Increasing value and reducing waste: addressing inaccessible research.^15^ Reducing waste from incomplete or unusable reports of biomedical research.^16^
**Point of view (2014)**
This series related to an article published by The Lancet in 2009: Avoidable waste in the production and reporting of research evidence.^17^

**Figure 1:**
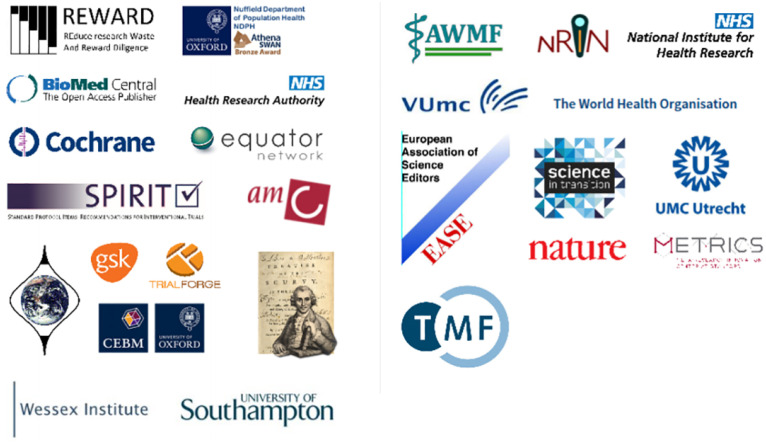
Editors engaged in REWARD – Reduce research Waste And Reward Diligence.

Furthermore, a campaign in 2014 that aimed to reduce waste within research, named **REWARD** (REduce research Waste And ***Reward*** Diligence), to which The Lancet subscribed, highlighted the central role of information specialists in helping to reduce waste within research. Journal editorial teams and funding bodies were brought into biomedical research centers to examine the rigor of research processes, assess the extent of uncertainty and identify relevant research that was in progress (Figure 1). When information specialists at the Cochrane Collaboration decided to rename their positions, as Trial Search Coordinators, this was in recognition of these evolving functions.^18^

## PRESS

This instrument was conceived and created with the aim of conducting and evaluating search strategies for syntheses of evidence. It can be used to initiate the bibliographic search process of any research and publication project with the aim of augmenting the quality and general coverage of research.

Table 3 presents an evidence-based verification list of guidelines for PRESS 2015.^8^

**Table 3:** Evidence-based verification list from the guidelines of PRESS 2015^8^

Translation of the research question	Does the search strategy correspond to the research question and PICO?
	Are the search concepts clear?
	Have many or few PICO elements been included?
	Are the search concepts too restrictive or too broad?
	Does the search recover many or few records? (Please show the number of occurrences per line.)
	Have unconventional or complex strategies been explained?
Boolean and proximity operators (these vary according to the search service)	Have Boolean or proximity operators been used correctly?
	Is the use of nesting with square brackets adequate and effective for the search?
	If NOT was used, is it likely that this has resulted in some undesired exclusion?
	Could the precision be improved by using proximity operators (for example, adjacent, near or within) or search for phrases instead of using AND?
	Is the width of the proximity operators appropriate? (For example, would adj5 get more variants than adj2?)
Subject headers (specific to the database)	Are the subject headers relevant?
	Are any relevant subject headers missing? For example, any previous index terms?
	Are any subject titles too broad or too narrow?
	Have the subject headers been exploded when necessary and vice versa?
	Have main titles been used (“starring” or restrictive in focus)? If so, is there adequate justification?
	Are subtitles missing?
	Are the subtitles attached to the subject headers? (Floating subtitles may be preferred.)
	Are the floating subtitles relevant and appropriately used?
	Have both subject headers and free-text terms (see below) been used for each concept?
Search for text words (free text)	Does the search include all spelling variants in free text (for example, British spelling versus American spelling)?
	Does the search include all synonyms or antonyms (for example, opposites)?
	Does the search capture relevant stems (i.e. is the stemming in the right place)?
	Is the stemming too broad or too narrow?
	Are the acronyms or abbreviations used appropriately? Do they pick up any irrelevant material? Have the complete terms also been included?
	Are the keywords sufficiently specific or too broad? Are too many or too few keywords used? Are stop words used?
	Have appropriate fields been searched? For example, was it appropriate to choose text word fields (.tw.) or all fields (.af.)?
	Are there any other fields to be included or excluded (specific to the database)?
	Should any long strings be divided into several shorter search declarations?
Spelling, syntax and line numbers	Are there any spelling mistakes?
	Are there any errors in the system syntax? For example, use of a stem symbol for a different search interface?
	Are there any incorrect combinations of lines or orphan lines? (In other words, are there any lines that are not mentioned in the final summary that might indicate an error in an AND or OR instruction?
Limits and filters	Have all the limits and filters been used appropriately and are they relevant for the research question?
	Have all the limits and filters been used appropriately and are they relevant for the database?
	Are any potentially useful limits or filters missing? Are the limits or filters too broad or too narrow? Could any limits or filters be added or removed?
	Have the sources for the filters used been cited?

This instrument provides descriptions of six elements for use as guidelines for librarians’ practices. Moreover, for editors, this can serve as an instrument for general methodological assessment of reviews.

It is important that editors and reviewers should adopt or establish peer review strategies for evaluating articles submitted for publication that involve input from a specialist librarian.^9^

The ideal is that all of this search process should be done at the start of the research, so as to avoid perpetuating errors, not just at the end of the study but throughout its course. There is no doubt that as soon as peer review practices for search strategies are implemented by editors and everyone involved in publication processes, authors will start to conduct searches with adequate criteria from the outset.

The idea would be to make it clear in the instructions for authors what criteria should be used for descriptions of methodologies and what instrument or combination of instruments the journal will be using for assessing the quality of studies that are submitted to it.

From the information in Table 1, a template of options for description can be created so that all studies submitted, and also those already conducted, can have better methodological descriptions and quality. MECIR and PRESS provide broad descriptions and rigor for use in all research. It is also important to note that PRESS will shortly be available in Portuguese.

There is a clear need to improve the adequacy of search strategies for systematic reviews and for reviews in general. The presence of a search specialist, with experience in developing strategies throughout the research process has become essential for ensuring transparency and reproducibility of research methods, thus benefiting the quality of the reviews produced.

It is important that the reviewer using the search strategy and the information specialist who designed the strategy should be supported by a national forum for search specialists and should have access to teams that could review their strategies. Furthermore, they should also use the use the verification list of PRESS, which summarizes the main potential errors made in search strategies.^9^

All efforts exerted towards improving the quality of all research and reviews are valid.

With the material that is made available, along with the tools and instruments, the next step is to work put a route along which editors can better assess search strategies that are submitted for publication.
